# Orofacial quantitative sensory testing: Current evidence and future perspectives

**DOI:** 10.1002/ejp.1611

**Published:** 2020-06-17

**Authors:** Fréderic Van der Cruyssen, Loes Van Tieghem, Tomas‐Marijn Croonenborghs, Lene Baad‐Hansen, Peter Svensson, Tara Renton, Reinhilde Jacobs, Constantinus Politis, Antoon De Laat

**Affiliations:** ^1^ Department of Oral & Maxillofacial Surgery University Hospitals Leuven Leuven Belgium; ^2^ OMFS-IMPATH Research Group Department of Imaging and Pathology Faculty of Medicine University Leuven Leuven Belgium; ^3^ Department of Oral Health Sciences KU Leuven and Department of Dentistry University Hospitals Leuven Leuven Belgium; ^4^ Section of Orofacial Pain and Jaw Function Department of Dentistry and Oral Health Aarhus University Aarhus Denmark; ^5^ Scandinavian Center for Orofacial Neurosciences (SCON) Aarhus University and Malmö University Aarhus Denmark; ^6^ Department of Oral Surgery King’s College London Dental Institute London UK; ^7^ Department of Dental Medicine Karolinska Institutet Stockholm Sweden

## Abstract

**Background and objective:**

Orofacial quantitative sensory testing (QST) is an increasingly valuable psychophysical tool for evaluating neurosensory disorders of the orofacial region. Here, we aimed to evaluate the current evidence regarding this testing method and to discuss its future clinical potential.

**Data treatment:**

We conducted a literature search in Medline, Embase and Scopus for English‐language articles published between 1990 and 2019. The utilized search terms included QST, quantitative, sensory testing and neurosensory, which were combined using the AND operator with the terms facial, orofacial, trigeminal, intraoral and oral.

**Results:**

Our findings highlighted many methods for conducting QST—including method of levels, method of limits and mapping. Potential stimuli also vary, and can include mechanical or thermal stimulation, vibration or pinprick stimuli. Orofacial QST may be helpful in revealing disease pathways and can be used for patient stratification to validate the use of neurosensory profile‐specific treatment options. QST is reportedly reliable in longitudinal studies and is thus a candidate for measuring changes over time. One disadvantage of QST is the substantial time required; however, further methodological refinements and the combination of partial aspects of the full QST battery with other tests and imaging methods should result in improvement.

**Conclusions:**

Overall, orofacial QST is a reliable testing method for diagnosing pathological neurosensory conditions and assessing normal neurosensory function. Despite the remaining challenges that hinder the use of QST for everyday clinical decisions and clinical trials, we expect that future improvements will allow its implementation in routine practice.

## 1 INTRODUCTION

For patients with sensory neuropathy, qualitative sensory testing (QualST) is the most commonly used method in clinical consultations, and quantitative sensory testing (QST) is purported to be useful for phenotyping (Forstenpointner, Otto, & Baron, 2017). Notably, both QST and QualST are considered to be subjective, and many authors recommend objective sensory tests for neuropathy assessment (Teerijoki‐Oksa et al., 2019). Nerve conduction tests such as somatic sensory evoked potentials, blink reflex, sudomotor and other reflex tests provide the most objective and repeatable measures as they exclusively assess the integrity of a few neural pathways (Dyck, 1991). However, they are not able to assess patient's symptoms and experience of their neuropathy, which is arguably the most important aspect to evaluate when the goal is treatment (Dyck, 1991). Thus, increasing attention has been focused on QST, and an ever‐growing body of published evidence supports its value. Orofacial QST has lifted off in the last 30 years and since the task force report on orofacial QST by Svensson et al. in 2011, many new insights have emerged (Svensson et al., 2011). We hope to bring an update of the literature in orofacial QST as this is lacking from the current literature.

In the present narrative review, we aimed to critically review existing evidence about QST in the orofacial area, to reflect on shortcomings and to elucidate future perspectives. Readers should be able to understand the fundamentals, strengths and pitfalls of orofacial QST after reading this paper. In addition, several pertinent questions have been raised. Does QST add to our clinical decision making? Does it correlate with specific diseases or pain syndromes and their severity? Does it influence our treatments? And what is its diagnostic value? To answer these questions, we first must establish basic information about QST. We will address the following questions. What is QST? How is it performed? Are the measurements and parameters for the orofacial area reliable and relevant? Can we diagnose and differentiate different pathologies? What factors influence outcomes? And does QST offer added value compared with other diagnostic aids? These questions will be answered using the most recent literature wherever possible.

## 2 METHODS

We performed a scoping literature search in Medline, Embase, Web of Science and Scopus using the following search terms: QST, quantitative, sensory testing and neurosensory. These terms were combined using the AND operator with: facial, orofacial, trigeminal, intraoral and oral. We included all English‐language articles published between January 1990 and January 2018. Articles were selected based on title and abstract screening, followed by full‐text analysis. We also performed manual screening of reference lists and the grey literature to identify other relevant articles.

## 3 DISCUSSION

**Figure 1:**
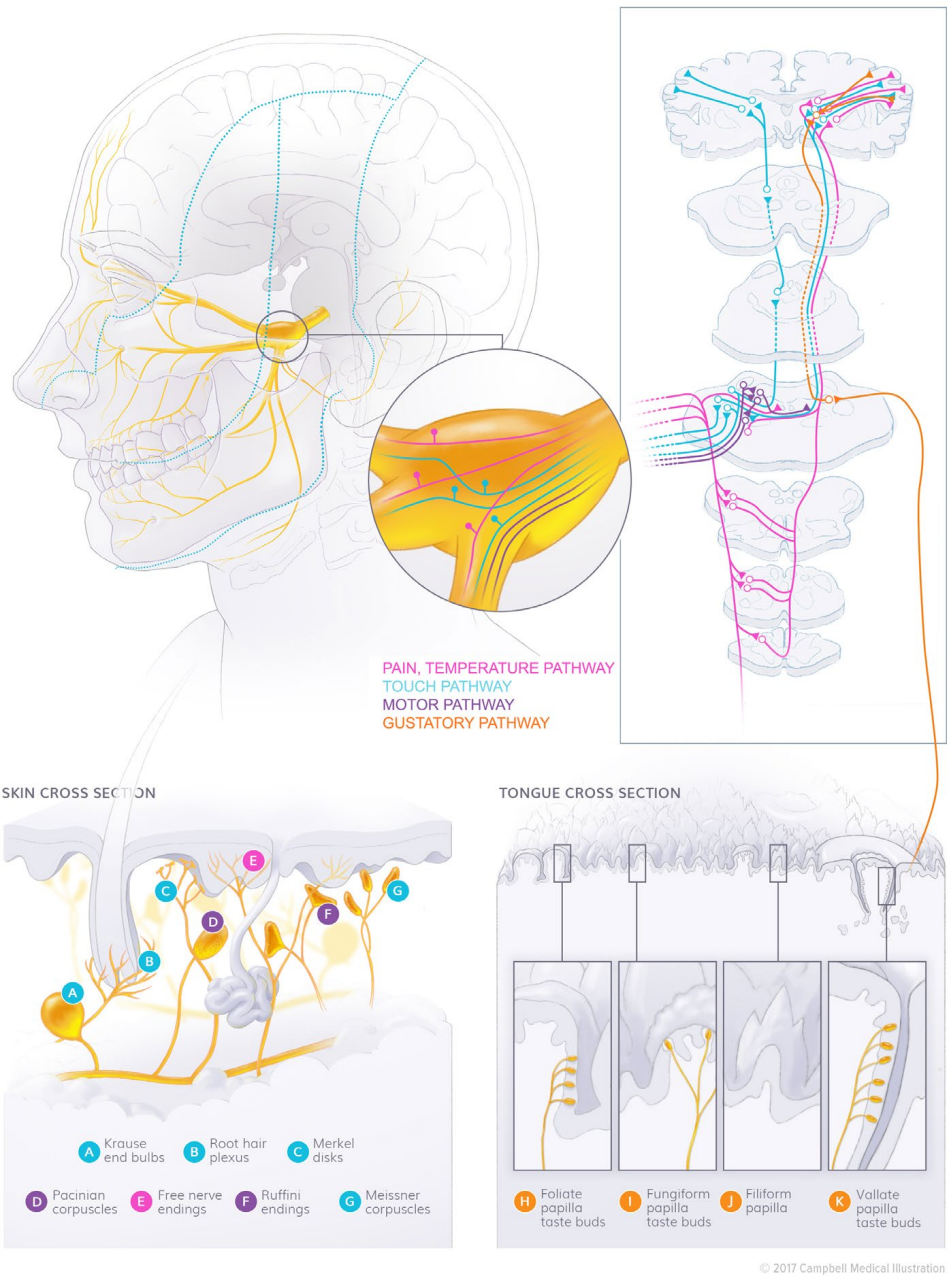
Trigeminal sensory and motor pathways. Sensory input from the orofacial area is carried through the trigeminal ganglion toward the trigeminal nuclei. There, the peripheral afferent neurons synapse with their secondary neuron, and convey sensory information through the thalamus towards the somatosensory cortex. Specialized receptors are found in the orofacial skin, mucosa, gingiva, tongue, periodontal tissues, joints and muscles

Before discussing diagnostic tools for detecting neurological disorders in the orofacial region, a thorough understanding of normal functioning and trigeminal neurophysiology is required. Multiple books provide an overview of this broad topic; however, our understanding of complex trigeminal neurophysiology and the various orofacial functions is still at an early stage. Our group has previously reviewed trigeminal neurophysiology (Svensson et al., 2011; Van der Cruyssen & Politis, 2018). Trigeminal pathways carry information for tactile and thermal stimuli, taste and nociception, as well as motor fibres (Figure 1). Understanding these pathways and their functions is important for interpreting clinical pathology and QST findings.

### 3.1 What is QST?

QST is performed with the goal of diagnosing and differentiating underlying pathophysiological somatosensory mechanisms based on subsets of responses. QST can differentiate multiple modalities of neurosensory disturbance—including mechanical or thermal allodynia, hyperalgesia, hypoesthesia, anaesthesia and disturbances of touch and directional sense. The recognition of different patterns that correlate with specific underlying mechanisms can lead to phenotyping, which may, in turn, guide adjustments of therapy. Several instruments have been developed for measuring neurosensory disturbances, including von Frey monofilaments, pressure algometers and thermal probes. In this review, we will introduce these modalities and discuss their practical use in psychophysical experiments.

QST is the term used to describe the application of quantitative methods to conduct research on the somatosensory nerve system (Mücke et al., 2016). The characteristics of the applied stimuli are defined (the modality, location, size of the contact area, duration, frequency and intensity). But, in contrast with QualST, the patient's response is measured quantitatively. QST is considered a psychophysical test because responses are subjective to the patient's perception and can be verbal or nonverbal (Schipper & Maurer, 2017). This is one advantage of QST over electrophysiological tests that do not consider the patient's perception of stimuli. Other advantages of QST include its non‐invasive nature, and its potential to evaluate the smaller A‐delta and C fibres, which cannot be tested using routine electrophysiological tests, such as somatosensory evoked potentials or electroneurography.

Disadvantages of QST include that it cannot be used to localize lesions in the neurological pathway towards the cortex, as well as the requirements that patients cooperate and understand the tasks and questions (Benzon, Fishman, Liu, Cohen, & Raja, 2011). It remains unclear whether QST actually reflects the patient experience. Certain aspects are not assessed by QST such as extent of the affected neuropathic area, paresthesia or spontaneous neuralgia (Walk et al., 2009). These symptoms may hold equally important information in diagnosis and in determining a management strategy. Additionally, the researcher must be trained in QST, and the method requires an environment that allow for quiet and methodical evaluation (Nothnagel et al., 2017). The required equipment is expensive, especially if the researcher wishes to carry out thermal sensory testing.

### 3.2 Methods of performing QST

Several methods can be used to vary the utilized stimuli, to assess the patient's responses to them. Some methods are better suited for use with specific stimuli, and methods can be combined in a battery of testing—for example, in the German Research Network on Neuropathic Pain (DFNS) QST protocol (Rolke et al., 2006). A recently published taskforce report on somatosensory assessment of the orofacial area provides guidelines for orofacial QST and future directions (Svensson et al., 2011). Here, we describe several commonly used psychophysical paradigms.

#### 3.2.1 Method of levels

In “method of levels” testing, a repetitive static stimulus is applied with the intensity and duration adjusted based on the response to the previous stimulus (Mücke et al., 2016). The limit is defined as the stimulus intensity at which 50% of stimuli are detected, producing an S‐like stimulus–response graph. The CASE IV system (WR Medical Electronics Co.) applies this testing method using the “just‐noticeable difference” (JND). If the participant perceives the stimulus, less‐intense stimuli are applied until the stimulus is no longer perceived, and vice versa. A participant who perceives level zero stimulus is considered hypersensitive, while one who does not perceive level 25 is considered insensitive. When small differences are used, this technique enables very precise level detection (Lue, Shih, Lu, & Liu, 2017). Additionally, this method has low interest variability and, thus, has relatively good reproducibility; however, it is time‐consuming and can lead to sensitization (Meier, Berde, DiCanzio, Zurakowski, & Sethna, 2001). Notably, heat pain thresholds cannot be determined using this method because tissue damage is possible, and respondents may anticipate the next stimulus by prematurely indicating a positive or negative response (Lue et al., 2017; Moloney, Hall, & Doody, 2012).

**Figure 2:**
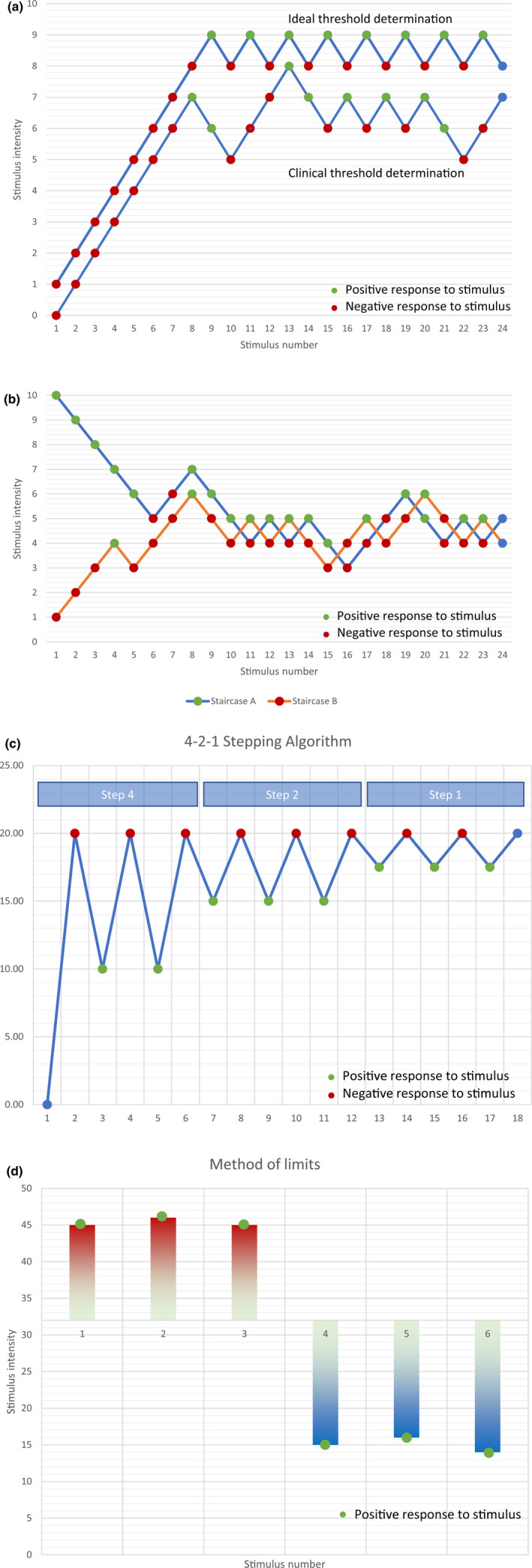
(a) Staircase method for level determination. A low or high stimulus intensity is chosen and is raised or lowered depending on the patient's response, until a positive response is given. The sequence is then reversed until a negative response is provided, and so on. After a predetermined number of stimuli, the average threshold is calculated. An ideal threshold is illustrated where the variation around the level is minimal and the patient's response is unequivocal versus a clinical situation, which is more in line with reality (b) Randomized staircase method, in which two staircases are combined and the utilized staircase stimulus is randomly selected. (c) The 4–2–1 stepping algorithm, in which an ever‐decreasing stimulus intensity is used to determine a threshold. (d) The method of limits determination, in which a continuously increasing or decreasing stimulus is applied until reaching a predetermined cue. For example, heat pain thresholds and cold pain thresholds are determined with this method

Selection of the different levels can be performed in several ways. In the forced‐choice method, the patient is given two or more response options, and must commit to an actual answer. Examples are the temporal forced‐choice method where a stimulus is applied in a certain time window or not. The patient must then indicate the time window in which the stimulus was administered. Another example is the spatial forced‐choice method. Here, the patient must choose between two presented probes and indicate which one was the predetermined stimulus (Chong & Cros, 2004). This technique is time‐consuming, and performance may decline because the participant becomes bored. To overcome this challenge, another method has been developed: the 4–2–1 stepping algorithm (Dyck, Zimmerman, et al., 1993). Unlike the forced‐choice method, the 4–2–1 stepping algorithm begins with a middle‐level stimulus and progresses via a stepwise approach based on the patient's responses. When the patient gives a consistent positive response to the applied stimulus, the stimulus is decreased in a stepwise fashion dividing its intensity or, for example, the inter‐prong distance in case of two‐point discrimination, narrowing the range in determining the final threshold level (Dyck, 1993). This method shows good inter‐rater and intra‐rater reliability when used for tactile threshold determination and for two‐point discrimination (Dyck, O'Brien, et al., 1993; Snyder, Munter, Houston, Hoch, & Hoch, 2016; Wikstrom & Allen, 2016). Lastly, the staircase method starts with a stimulus of high intensity (or low intensity), which is then lowered (or raised) until the patient no longer perceives the stimulus (or begins to perceive the stimulus). Then the staircase is reversed until a new positive (or negative) response is given, which then triggers another reversal. This method is used in the DFNS protocol for determination of tactile and pain thresholds. A modification of this technique involves the use of two staircases: one starting with a high intensity and the other starting with a low intensity. The alternation between staircases can be randomized to reduce both participant and examiner bias (Cornsweet, 1962). An overview of these methods is provided in Figure 2.

#### 3.2.2 Method of limits

With the “method of limits,” stimulus intensity is raised or lowered until it is perceived or no longer perceived, respectively (Mücke et al., 2016). The threshold is marked by a button or a verbal cue stopping further stimulation. This can be repeated several times to determine an average threshold to the stimulus. The utilized stimuli are considered dynamic, and are less time‐consuming to conduct than those applied in the method of levels (Lue et al., 2017). The method of limits can be used to determine tactile detection thresholds, thermal heat and cold noxious and innocuous thresholds, vibration, and deep pain thresholds. Thresholds are determined using this method in the DFNS protocol (Rolke et al., 2006). Intensity must be slowly increased with a standardized ramp (e.g., 1°/second) or decreased to minimize the influence of reaction time. This method is subject to habituation (Palmer & Martin, 2005).

#### 3.2.3 Method of adjustment

The method of adjustment allows patients to adjust the stimulus intensity themselves (Pelli & Farell, 1996). An example in orofacial QST could be the application of a thermode in the mental area. Next, the patient is given a control button and asked to raise the temperature until the heat pain threshold is reached. This limits the patient's loss of interest. However, this method is rarely used because, other than electrical stimuli, most stimulus modalities are difficult to apply in this manner. The authors could not identify any application of this method in orofacial QST.

#### 3.2.4 Suprathreshold intensity rating

Suprathreshold intensity rating involves the application of several known stimuli with intensities above the detection threshold (Snyder, Sims, & Bartoshuk, 2015). The participant scores the intensities on a numerical rating scale (NRS) or a visual analogue scale (VAS). The data can be used to draw a stimulus–response curve. It is important to define the lower and upper limits—for example, when measuring pain, zero would indicate no pain and 100 would indicate the worst imaginable pain. The magnitude estimation scale is constructed by defining a standard stimulus. The patient scores the next stimulus in relation to the standard modulus. One disadvantage of this scale is that calculation of the mean is influenced by the randomly determined first score. Previous studies demonstrated that pain sensitivity, which can be measured using this method, decreases with age (Blankenburg et al., 2010; Hirschfeld et al., 2012). This demonstrates the importance of having reliable reference tables stratified for sex and gender.

#### 3.2.5 Mapping

In mapping, a thermoroller or marching needle technique is used to identify areas having the same somatosensory properties. However, intraoral application remains challenging. Since it is easy to perform, the mapping technique is often used in QualST (Michael Miloro, 2015; Renton, Thexton, Crean, & Hankins, 2006; Robinson, Smith, Johnson, & Coppins, 1992). In a previous study, it was suggested that the affected surface percentage of the trigeminal dermatome may indicate whether nerve injury will be permanent (Renton & Yilmaz, 2011).

### 3.3 Different modalities and stimuli

Orofacial receptors and their nerve fibres can be clinically tested to assess the integrity of different fibre types. Several stimuli have been designed to assess these different modalities (Jacobs, Wu, Goossens, et al., 2002). Most stimuli trigger multiple receptors at once. The numbers and types of receptors that are recruited may influence the patient's perception, and thus response, to the stimuli (Abraira & Ginty, 2013; Dubin & Patapoutian, 2010; Jones & Berris, 2002).

#### 3.3.1 Mechanical stimulation

Non‐painful tactile stimuli are conveyed via A‐beta fibres, which can be tested using monofilaments (Landerholm & Hansson, 2011). The filament is placed perpendicularly to the tested surface for 1–2 s until it bends and is then kept in place for 2 additional seconds before the stimulus is removed. The filaments bend with pressure forces ranging from 4 mg to 300 g. Von Frey originally used horse hairs, while Semmes and Weinstein used nylon and further standardized these hairs (e.g., the Weinstein Enhanced Sensory Test and Semmes–Weinstein Filaments) (Weinstein, 2012). Other hairs have been developed to further optimize these filament tests. Rather than nylon, OptiHair2 uses glass fibres with rounded tips that make them more durable (Somedic, Schriesheim, Germany). Most filaments are calibrated and express a logarithmic relation between filament diameter and force. Thus, a scale is often used to convert the coded filaments into grams and force per area or millinewtons (mN), which can then be translated into residual sensory function for clinical interpretation.

The traditional Semmes–Weinstein filaments are not useful for intraoral testing because their properties change in humid environments, and their design is not optimized for intraoral use (Haloua, Sierevelt, & Theuvenet, 2011). However, reliable light touch thresholds of the anterior oral mucosa using these filaments have been reported (Jacobs, Wu, Van Loven, et al., 2002). In this study, thresholds measured using the staircase method did not differ significantly from the ascending and descending method. Preferred options for intraoral testing include optic glass fibre filaments with forces from 0.125 to 512 mN, with a rounded tip and a 0.5‐mm cross‐sectional diameter. Another option is Cheung–Bearelly monofilaments, which can be constructed by researchers themselves and are easily utilized. However, their stimulus intensity is not calibrated, making comparison with other QST research difficult (Bearelly & Cheung, 2017). In the future, the use of airflow stimulation could overcome some of the technical issues encountered with intraoral filament use (Tsalamlal, Ouarti, & Ammi, 2013). Dynamic tactile stimulation can be tested using a cotton swab or toothbrush, and is helpful for the detection of allodynia and determining directional sense (Renton & Van der Cruyssen, 2019; Svensson et al., 2011).

#### 3.3.2 Two‐point discrimination

Two‐point discrimination is the minimum separation that a patient can detect between two simultaneously applied tactile stimuli, ideally having the same intensity (Sang‐Yeun, Kim, Kim, & Kim, 2017). This phenomenon depends on peripheral innervation density (Sang‐Yeun et al., 2017). In the orofacial area, the distance varies between 2 and 30 mm. Intraorally, this technique is frequently applied on the tongue tip and the vermillion. It can also easily be performed on the anterior oral mucosa. One study used self‐constructed calibrated pressure probes and found an overall mean two‐point discrimination of 9.2 mm for the oral buccal mucosa of anterior upper jaw but they did not assess other orofacial areas (Jacobs, Wu, Van Loven, et al., 2002). Another study reported normative data on several trigeminal areas, and included information regarding sex, site and stimulus‐dependent values (Sang‐Yeun et al., 2017). The results showed that women have a higher discriminative ability than men, and that the tongue tip and lower lip are more sensitive than the cheek and forehead. Gingival and mucosal surfaces were not analysed in that study (Sang‐Yeun et al., 2017). Flexible calibrated filaments can be used to overcome inaccuracies and variability caused by the application of different stimulus intensities when using a two‐point discrimination device. Isobaric pressure meters have also been suggested as a means of overcoming these inaccuracies and variability (Manivannan, Periyasamy, & Suresh, 2015).

#### 3.3.3 Vibration

The Rydel–Seiffer tuning fork is the standard device currently used for testing vibration. This device works best when placed on thin skin–bone contact (Lai, Ahmed, Bollineni, Lewis, & Ramchandren, 2014). After the fork is snapped into motion, it is placed on the test area. The patient is asked to indicate when the vibration is no longer felt. The intersect between the apparent triangles indicated on the fork is recorded by using an arbitrary scale from 0 to 8 (Panosyan, Mountain, Reilly, Shy, & Herrmann, 2016). Application on the tongue is difficult to standardize as it is not supported by a bony floor. Additionally, the tuning fork design cannot easily be used for intraoral testing of gingival areas due to the required angulation. Nevertheless, intra‐oral vibration thresholds have been reported (Pigg, Baad‐Hansen, Svensson, Drangsholt, & List, 2010). Vibration can be similarly tested by electronic vibrators that have adjustable frequency, amplitude and pressure (Jacobs, Wu, Van Loven, et al., 2002). Different amplitudes induce the activation of different mechanoreceptors. A vibrating electric toothbrush is a viable alternative device that can be used for intra‐ and extra‐oral vibro‐tactile testing (Nixdorf, Hemmaty, Look, Schiffman, & John, 2009). However, it remains unclear whether anything similar to allodynia exists with regards to vibration.

#### 3.3.4 Pinprick

To determine mechanical pain thresholds, researchers use pinprick stimulators, which are usually thicker (and sometimes electric) von Frey filaments, or force‐calibrated pins or needles. Standardized blunt needles with a 0.25‐mm diameter and a weight range from 8 to 512 mN are used, and the shape, size and angulation affect the pain threshold. Modified dental probes can also be used intraorally.

#### 3.3.5 Deep pressure

Both simple and more sophisticated pressure algometers are available, and several types have been described for intraoral use (Davenport, 1969; Ogawa, Ogimoto, Sumiyoshi, & Koyano, 2003; Ogawa et al., 2011; Ogimoto, Ogawa, Sumiyoshi, Matsuka, & Koyano, 2002). For deep pressure measurements, pain and tolerance thresholds are sought. For orofacial testing, the study by Pigg et al. applied probe diameters of 4.8 mm for intraoral use and 1.1 cm for extraoral use (Pigg et al., 2010). The pressure can be changed at different rates, with a recommended rate of 50 kPa/s, and three separate measurements should be performed at 1‐min intervals (Pigg et al., 2010). A previous study determined intraoral pressure‐pain thresholds, and reported high variability between different tested sites, as well as disproportionate modulation when pre‐loading different sites (Ogawa et al., 2003; Ogimoto et al., 2002).

#### 3.3.6 Thermal stimulation

Devices for thermal stimulation, guided by A‐delta and C fibres, are evolving. Early testing was performed using copper and aluminium rods with diameters of up to 1 cm, and a variant thereof, comprising four discs made of different materials (Minnesota Thermal Disks, WR Medical Electronics Co.). Investigations with these devices provided the first insights into thermal topographical variation. Tests were also performed using thermal rollers that are cooled or heated in a water bath (Marchettini, Marangoni, Lacerenza, & Formaglio, 2003). These materials are still used today, but mainly for qualitative research. Drawbacks include difficulty controlling the temperature, and the fact that they exert mechanical stimuli in addition to thermal stimuli (Marchettini et al., 2003).

The use of thermal bars and discs was replaced by thermodes. Thermal contact stimulators were designed that enabled the application of precise stimuli to the skin or mucosa. These stimulators comprise a thermoelectric heating and cooling element (according to the Peltier principle) and have a contact surface of up to 10 cm^2^ (Pathway, Medoc, Ramat Yishai, Israel). Investigation of the orofacial region requires smaller contact areas (1–4 cm^2^) to test the different dermatomes of the trigeminal nerve, which can be too small to test with regular probes or instruments (Svensson et al., 2011). However, the use of different contact areas will recruit different receptor fields such that the results of QST assessment may also change (Jones & Berris, 2002). This was proven for orofacial thermal thresholds, where an increasing stimulus area was associated with spatial summation for warm and heat pain thresholds, but not cold detection thresholds (Pigg, Svensson, & List, 2011). Intraoral probes are now available for several commercial systems. These systems enable linear temperature changes, as required for the “method of limits,” as well as a rapid return to baseline temperature. However, the disruptive effect of mechanosensation is still present. Thermodes with a Peltier element and water‐cooling system are still used, but are now computer‐based, such as the Thermal Sensory Analyzer (TSA) II (Medoc, Israel), Pathway (Medoc, Israel) and Modular Sensory Analyzer (MSA, Somedic, Sösdala, Sweden).

The second important group of currently used equipment includes radiant heat or laser stimulators (Franz et al., 2012; Svensson, Bjerring, Arendt‐Nielsen, & Kaaber, 1992). Light energy is absorbed by the tissue surface, causing a rise in temperature. This technique has the major advantage that no tactile afferent fibres are activated. The disadvantages are that the generated skin temperature is not monitored, only heat tests can be performed (not cold), there is a risk of tissue damage, and the system is expensive and requires technical maintenance (Franz et al., 2012). Most lasers (including argon, CO_2_, Nd‐YAG and thulium‐YAG lasers) can be used intraorally, but sometimes they can only be applied in the anterior region because the rays have to pass through articulating arms. A recently launched new generation of laser stimulators, termed diode lasers, are more stable, smaller and cheaper, and are thus promising for QST (Moeller‐Bertram, Schilling, Bačkonja, & Nemenov, 2013).

In addition to the above‐described complex devices for thermal stimulation, there is also a simple test that estimates central sensitization. An ice cube is held in the mouth, and then removed, and examiners check for the presence of an “after‐sensation” (Zhu et al., 2019). Alternatively, an ethyl chloride canister can be used to cool a dental probe, which can be applied extraorally or intraorally (Ahlquist & Frmzta, 1994). Recent research has focused on dynamic QST, such as conditioned pain modulation (CPM) to assess endogenous pain inhibition (Mackey, Dixon, Johnson, & Kong, 2017). One method of CPM assessment involves the use of a painful thermal stimulus (e.g., an ice‐cold water bath) as a conditioning stimulus. Additional research will be needed to improve standardization of CPM protocols, and to assess its clinical implications in orofacial pain (Kennedy, Kemp, Ridout, Yarnitsky, & Rice, 2016).

#### 3.3.7 Other variations

There are several other variations of techniques. First, oral perceptual abilities can be assessed based on stereognosis—the recognition of form or shape in the oral cavity (Ahmed, Hussain, & Yazdanie, 2006). Such assessment can be beneficial for both planning and predicting future outcomes of any treatment modality in the orodental region. One group has performed two studies to assess the use and reliability of grating domes (Stoelting, Wood Dale, USA) (Van Boven & Johnson, 1994a, 1994b). They used several methods to investigate patients who underwent orthognathic surgery, and found that the grating orientation test was a better predictor of neurosensory deficit than the patient's subjective report (Van Boven & Johnson, 1994a, 1994b). However, another study group reported that postoperative grating dome testing did not correlate well with intraoperative nerve damage (Teerijoki‐Oksa et al., 2003). These tests require inputs and integration from multiple receptors, synapses, nuclei and (sub‐)cortical areas, which may be more clinically meaningful than assessing a single receptor response.

Second, occlusal sensitivity can be transduced via mechanoreceptors embedded in the periodontal ligaments (Jacobs, Bou Serhal, & van Steenberghe, 1998; Jacobs & van Steenberghe, 1994). Notably, pulpal, muscular and articular receptors also contribute to occlusal sensitivity, and osseoperception has been described around implants lacking a periodontal ligament (Jacobs & Van Steenberghe, 2006).

Third, in dentistry, pulpal sensitivity and vitality testing are commonly used to assess pulp vitality in cases of periodontal disease and caries, which helps guide treatment decisions (Dabiri et al., 2018). Pulp sensitivity is also conveyed via the trigeminal afferents, which can be evaluated by applying cold rods against the tooth (Odontotest, Fricar, Zurich, Switzerland) or using electric pulp‐testing devices (Chen & Abbott, 2009). These tests have varying ranges of sensitivities and specificities, and are qualitative in nature because the patient simply indicates whether they perceive the stimulus.

Lastly, blink and muscle reflexes can be evaluated to assess the integrity of the neuro‐muscular pathways, and to thus partly assess central processing and integration with different cranial nerves (Aramideh & Ongerboer De Visser, 2002; Brodin, Türker, & Miles, 1993; Cruccu et al., 2005). One study assessed blink reflexes in atypical odontalgia patients compared to healthy individuals. They revealed that the patients showed a reduced late blink reflex signal (Baad‐Hansen, List, Kaube, Jensen, & Svensson, 2006).

#### 3.3.8 Extensive QST protocol

In 2006, the German Research Network on Neuropathic Pain (DFNS) compiled a “QST battery” of seven tests, including 13 parameters: cold detection threshold (CDT), warm detection threshold (WDT), paradoxical heat sensation (PHS), thermal sensory limen (TSL), cold pain threshold (CPT), heat pain threshold (HPT), mechanical detection threshold (MDT), mechanical pain threshold (MPT), mechanical pain sensitivity (MPS), dynamic mechanical allodynia (DMA), wind‐up ratio (WUR), vibration detection threshold (VDT) and pressure pain threshold (PPT) (Rolke et al., 2006; Vollert et al., 2016). These parameters can be measured intraorally and extraorally, and represent almost all sensory modalities. A z‐transformation is performed to eliminate the different units used to describe the various parameters, allowing easy comparison. The DFNS protocol is currently used worldwide, and its reproducibility and reliability are considered sufficient for skin and intraoral measurements (Huge et al., 2011; Pigg et al., 2010). Some concerns have been raised regarding the different statistical methods used to assess test–retest reliability in QST research, and recommendations for future research have been provided (Werner, Petersen, & Bischoff, 2013).

### 3.4 Factors that influence QST

Factors that influence the final QST results can be separated into intrinsic and extrinsic factors. The intrinsic factors are due to differences in somatosensory function. Extrinsic factors are those factors that influence the QST equipment and its application. Extrinsic factors and how they may influence QST results have been described above. In the next paragraphs, we will discuss intrinsic factors. The available QST data suggest that the face is the most sensitive region of the body (Magerl et al., 2010). Sensitivity decreases in the orofacial posterolateral direction, and gingival sensitivity is lower compared to in the tongue and face (Baad‐Hansen et al., 2013b; Green, 1984; Pigg et al., 2011). Studies also report sex differences in various QST parameters, with women clearly having lower pain thresholds than men for most stimulus modalities (Ahn & Kim, 2015; Fillingim, King, Ribeiro‐Dasilva, Rahim‐Williams, & Riley, 2009). QST seems to be influenced by age, but to only a limited degree in the face (Blankenburg et al., 2010; Dyck, 1986). Ethnicity also plays a role in influencing QST (Yang et al., 2013). Moreover, it is possible to modulate the trigeminal somatosensory function, such as in conditioned pain modulation (Oono, Baad‐Hansen, Wang, Arendt‐Nielsen, & Svensson, 2013).

QST could be further influenced by treatments that our patients undergo. One randomized double‐blinded controlled trial assessed the effect of low‐level laser therapy (LLLT) on QST and pain ratings in patients undergoing orthodontic treatment. It was shown that patients treated with LLLT had lower pain ratings and higher CDT, WDT, CPT, HPT thresholds indicating a treatment effect that is measurable with QST (Wu et al., 2018). In other fields, the effect of analgesics has shown to affect QST results and vice versa QST may predict the analgesic response but more research will be needed to further substantiate these statements (Grosen, Fischer, Olesen, & Drewes, 2013; Wilder‐Smith, Tassonyi, Crul, & Arendt‐Nielsen, 2003).

Researchers have established normative reference values for the QST parameters assessed by the DFNS protocol, and have stratified the results according to age, sex and body region (Magerl et al., 2010). For the orofacial area, reference values are available for the second division of the trigeminal nerve (V2) and intraorally (Baad‐Hansen et al., 2013a; Magerl et al., 2010; Pigg et al., 2010). No normative datasets have been published for the ophthalmic and mandibular divisions of the trigeminal nerve, nor are there any normative intraoral datasets for the lingual, maxillary and inferior alveolar nerves, stratified according to age and sex. This lack of data limits the possibility of determining whether results deviate from the standard, and complicates clinical decision making, although some knowledge may be obtained by comparisons between an affected site and its mirror‐image contralateral site. Moreover, only limited research has investigated other factors that may influence orofacial QST; therefore, conclusions must be extrapolated from data from other body regions. This could be problematic since the orofacial area has unique characteristics that must be considered when performing QST. Notably, the innervation density and fibre ratio shifts from the forehead to the perioral tissues (Nolano et al., 2013). Intraoral QST can be difficult to obtain due to limited access, saliva may change stimulus transduction and complicate stimulus application, and tissue elasticities differ between test areas. For example, deep pressure pain thresholds are markedly lower at the tongue compared to a mucosal surface overlying the jaw bones (Pigg et al., 2010). Finally, it may be important to rethink the design of future clinical and experimental trials using intraoral QST as high variability between and within subjects at different levels needs to be accounted for and may substantially influence the required sample size (Moana‐Filho et al., 2017). Further research is needed to fill this knowledge gap, and overcome these issues.

### 3.5 Correlation with pathogenesis and severity

QST can provide indirect insights into the underlying mechanisms of pathophysiology, as has been demonstrated for polyneuropathy, postherpetic neuralgia and post‐traumatic nerve injuries (Freeman, Baron, Bouhassira, Cabrera, & Emir, 2014). In each of these pathologies, specific QST patterns are dominant, and may thus correlate with the underlying pathophysiology, which would allow easy differentiation between these entities (Vollert et al., 2017). A previous study identified a significant interaction between treatment with oxcarbazepine and the irritable or non‐irritable phenotype, regardless of the cause (Sindrup et al., 2014). This indicates that QST can play a role in elucidating the common pathophysiological pathways of diseases and in guiding treatment choices, thus supporting the field of personalized medicine.

To further investigate the correlation of QST with pathogenesis, we need a more thorough understanding of pathophysiology and disease progression, and of how QST results change over time. Patients undergoing orthognathic surgery may be a good clinical model for assessing longitudinal changes. These patients undergo elective surgery, which allows for baseline QST acquisition, and they often have a standardized follow‐up protocol. Due to the position of the inferior alveolar nerve during a sagittal split osteotomy, most patients experience postsurgical neurosensory disturbances but typically recover in the following months (Alolayan & Leung, 2017). In this setting, QST profiling, randomization and treatment effects could be analysed and followed‐up. One study analysed the correlation of intraoperative nerve damage with postoperative QST and electrophysiological findings, revealing a large variation in the sensitivity and specificity of the various modality test methods (Teerijoki‐Oksa et al., 2003). They suggested using a combination of nerve conduction study, touch detection thresholds and thermal QST to achieve adequate sensitivity and specificity. These results were confirmed in their more recent work (Teerijoki‐Oksa et al., 2019).

### 3.6 Current and future roles of QST in clinical decision making

One randomized, double‐blind, placebo‐controlled trial evaluated the effects of oxcarbazepine in peripheral neuropathy patients with different sensory profiles, and reported the usefulness of stratifying patients into different profiles (in this case, irritable nociceptor versus non‐irritable nociceptor phenotypes) rather than according to aetiology (e.g., diabetic neuropathy versus postherpetic neuropathy) (Sindrup et al., 2014). Stratification by profiles yielded a lower number needed to treat and revealed a significant effect between treatment and phenotype. This indicates that cohorts in clinical trials should be stratified according to their baseline sensory profile rather than their underlying aetiology, and potentially enriched with patients most likely to respond to study drugs.

To date, no such trials exist in the orofacial domain. A large number of patients experience orofacial pain, which can be the result of many pathologies, some of which are difficult to differentiate. Orofacial QST could play an important role in identifying populations that would benefit from a tested drug. However, no such interventional studies using orofacial QST have been published. We further wonder whether orofacial QST could be used to evaluate treatment effects over time, and to identify whether the underlying pathophysiology is arrested. No published studies have assessed orofacial QST parameters as a follow‐up tool in orofacial pain patients.

### 3.7 Longitudinal QST

Orofacial QST is primarily used as a diagnostic tool. In *healthy* volunteers, QST has shown to be reliable over time (Hirschfeld et al., 2012). A recent study in 22 healthy volunteers showed reliable QST results over a 10‐week period, supporting the use of QST to assess changes over time in clinical trials (Nothnagel et al., 2017). Another study reported reliable QST results for touch and cold detection thresholds after orthognathic surgery at follow‐up times of 2 weeks, 3 months and 12 months (Teerijoki‐Oksa et al., 2004). Moreover, the touch detection thresholds showed an excellent correlation with patient‐reported subjective neurosensory disturbances. A multi‐centre study was conducted to assess the reliability of intra‐oral QST in atypical odontalgia and healthy controls. Atypical odontalgia patients showed more QST abnormalities than the healthy controls, and the QST results had a good‐to‐excellent correlation with QualST. Additionally, the authors reported fair‐to‐excellent inter‐rater and intra‐rater observations and test–retest reliability (Baad‐Hansen et al., 2013b, 2015). Finally, another study assessed only one modality, and demonstrated that QST was reliable within and between patients (Ogimoto et al., 2002). More research is needed to assess orofacial QST in measuring treatment response or disease.

### 3.8 Practical issues

The most frequently mentioned problem with QST is the assessment duration (Mücke et al., 2016). To assess one extraoral area and compare it with the contralateral side, the investigator and patient must spend about 1 hr on testing, depending on their understanding of the tasks and the need for a break. Intraoral testing entails a more difficult application and necessitates allowing jaw relaxation or swallowing between the tests such that this assessment takes about 1.5 hr. This is cumbersome and limits QST implementation in routine clinical practice.

Several studies have assessed the correlation between QualST and QST, looking for a means of obtaining reliable results more quickly. One study compared QualST and QST in patients undergoing local anaesthetic blocks, and found that both assessment methods correctly indicated sensory loss at the infraorbital and mental nerve at several time‐points after block administration compared with saline injection (Kothari et al., 2019). However, QualST did not detect a significant difference between 10 min and 2 hr after block anaesthesia, whereas QST revealed a return towards normal baseline stimulus perception at the 2‐hr interval. Notably, other studies have shown glaring discrepancies between QualST and QST results (Agbaje, De Laat, Politis, et al., 2017; Jääskeläinen, 2004; Teerijoki‐Oksa et al., 2003, 2019). Most studies report that qualitative (clinical) sensory testing has a high specificity and a low sensitivity (Agbaje, De Laat, Constantinus, Svensson, & Baad‐Hansen, 2017; Teerijoki‐Oksa et al., 2019). This indicates that these tests could be useful in the clinical setting to assist in making a differential diagnosis and can be performed to exclude the presence of neurosensory disturbances. Thus, QualST could be used as an initial screening tool to indicate whether further QST testing is required. Others nuance these findings and report moderate correlation between QST and QualST (Baad‐Hansen et al., 2013b). This indicates that more research may be needed to develop better or combined QualST methods and to compare these with QST to assess the usefulness in healthy and pathological cohorts. A combination of some QST parameters such as thermal and mechanical thresholds with other methods such as neurography could have an additional benefit on test duration (Teerijoki‐Oksa et al., 2019). Until now, we could not find any reports that charted this time aspect.

### 3.9 Alternative diagnostic tools

Few published studies have compared QST with other diagnostic methods. One previously mentioned investigation compared some QST modalities with nerve conduction studies (NCS) of the inferior alveolar nerve, reporting that NCS showed a higher sensitivity compared to QST or QualST (Teerijoki‐Oksa et al., 2019). Additionally, one study evaluated QualST with magnetic resonance neurography in 42 patients with nerve injury after molar extraction. The results showed that nerve calibre and signal intensity measured on MRI were moderately‐to‐well correlated with clinical sensory testing performed using spatial, tactile, thermal and pain thresholding (Dessouky, Xi, Zuniga, & Chhabra, 2018). Imaging could potentially play a more important role in the diagnosis of orofacial neuropathies in the future, but currently only fMRI and diffusion tensor imaging studies can provide functional information about neurophysiology and abnormalities (Martín Noguerol et al., 2019). Additionally, the trigeminal nerve has a very difficult trajectory, with a broad distribution of thin fibres surrounded by an extensive vasculature, complicating radiographic evaluation. Susceptibility artefacts may further complicate the assessment (Hirata et al., 2018). Further studies should compare QST and imaging findings, to determine their roles in clinical decision making.

## 4 CONCLUSION

Evidence concerning orofacial QST and its diagnostic value has markedly increased over recent years, demonstrating that QST is a reliable method for assessing neurosensory function under normal and pathological conditions. Translation of QST to clinical practice remains challenging due to several factors, and additional research is needed to enable differentiation between pathological entities. Integration of the entire QST battery, or the use of some QST parameters combined with other diagnostic tools, could further increase accuracy and support QST implementation in routine practice.
